# Supplementary material for: Infectivity of SARS-CoV-2 on Inanimate Surfaces: Don’t Trust Ct Value
Source: Int J Environ Res Public Health. 2022 Dec 19;19(24):17074. doi: 10.3390/ijerph192417074 (PMC9779331; doi:10.3390/ijerph192417074)
Supplement: Supplementary file 1 [file ijerph-19-17074-s001.zip › ijerph-2043754-supplementary.pdf]

Table S1 Viral reduction on SARS-CoV-2 contaminated surfaces measured by PCR

Specimen	method	RNA copies control[1]	RNA copies after procedure[1]	Log reduction[2]
1	time >2 h	3.42E+05	3.39E+05	0.004
	ozone open	3.42E+05	1.04E+04	1.518
	ozone locked	3.42E+05	3.32E+04	1.014
	UV 200 mJ/cm^2	3.42E+05	4.07E+04	0.924
	UV 50 mJ/cm^2	3.42E+05	1.12E+05	0.484
	alcohol	2.80E+05	1.24E+05	0.356
2	time >2 h	2.22E+04	2.06E+04	0.033
	ozone open	2.22E+04	3.24E+02	1.835
	ozone locked	2.22E+04	5.94E+02	1.572
	UV 200 mJ/cm^2	3.01E+04	2.87E+03	1.020
	UV 50 mJ/cm^2	3.01E+04	7.64E+03	0.596
	alcohol	3.51E+04	8.20E+03	0.632
3	time >2 h	1.82E+05	1.71E+05	0.029
	ozone open	1.82E+05	3.56E+03	1.709
	ozone locked	1.82E+05	1.84E+04	0.995
	UV 200 mJ/cm^2	1.82E+05	1.54E+04	1.073
	UV 50 mJ/cm^2	1.82E+05	4.64E+04	0.594
	alcohol	2.01E+05	1.32E+05	0.181
4	time >2 h	1.33E+04	1.33E+04	0.000
	ozone open	1.33E+04	2.45E+02	1.736
	ozone locked	1.33E+04	6.36E+02	1.321
	UV 200 mJ/cm^2	1.72E+04	2.53E+03	0.833
	UV 50 mJ/cm^2	1.72E+04	6.21E+03	0.443
	alcohol	1.91E+04	3.78E+03	0.704

[1]mean of 3 experiments with two surfaces each

[2]calculated as difference of the log_{10} from the mean of control and the log_{10} from the mean after procedure

Table S2 Viral reduction on *Φ*6 contaminated surfaces and Ct change

method	pfu control[1]	pfu after procedure[1]	Log reduction[2]	ΔCt[3]
time >2 h	1,07E+07	6,57E+06	0,213	0,230
ozone open	1,07E+07	<10	6,076	12,767
ozone locked	1,07E+07	<10	6,076	10,833
UV 200 mJ/cm^2	1,07E+07	9,67E+01	5,045	1,065
UV 50 mJ/cm^2	1,07E+07	3,34E+03	3,508	0,817
alcohol	1,07E+07	<10	6,076	-0,197

[1]mean of 3 experiments with two surfaces each

[2]calculated as difference of the log_{10} from the mean of control and the log_{10} from the mean after procedure

[3]calculated as difference of the mean Ct of 3 experiments between the values after procedure and the control
